# Infant Regulatory Problems and Subsequent Behavioral Difficulties: The Mediating Role of Parenting Stress

**DOI:** 10.3390/children13040494

**Published:** 2026-03-31

**Authors:** Ina Nehring, Daria Reitmeier, Anna Friedmann, Volker Mall, Michaela Augustin

**Affiliations:** 1 Social Pediatrics, School of Medicine and Health, Technical University of Munich, 81675 Munich, Germany; d.reitmeier@tum.de (D.R.); anna.friedmann@tum.de (A.F.); volker.mall@tum.de (V.M.); michaela.augustin@tum.de (M.A.); 2 German Center for Child and Adolescent Health (DZKJ), Partner Site Munich, 80337 Munich, Germany; 3 Department of Medicine, Pediatrics, HMU Health and Medical University Potsdam, 14471 Potsdam, Germany; 4 kbo-Kinderzentrum, Heiglhofstrasse 69, 81377 Munich, Germany

**Keywords:** crying, sleeping, feeding, children, parents, mediation

## Abstract

**Background/Objectives**: Infant regulatory problems (RPs) are at risk of persisting and can contribute to later behavioral difficulties. Parenting stress has been identified as a risk factor associated with child RPs, but its mediating role has rarely been investigated in this context. The aim of the study was (1) to investigate whether RP symptoms were related to subsequent infant RP symptoms/toddler behavioral and emotional problems (BEPs) between two pediatric check-ups in the first 3 years of life and (2) to investigate the potential role of parenting stress as a partial mediator in the association of infant RPs and subsequent RPs/BEPs. **Methods**: Using data from a German cohort study (CoronaBaBY), associations between infant RPs at baseline and RPs/toddlers BEPs at follow-up (around 8 months later) were analyzed. Parenting stress was included as a mediation variable into the model. **Results**: In total, 725 parent–child dyads were analyzed. Mean infant age was 5.0 months (SD = 3.4). Elevated RP symptoms at baseline significantly predicted infant RP symptoms and BEPs at follow-up. Parenting stress at baseline significantly predicted feeding problems and BEPs at follow-up. Parenting stress partially mediated the associations between baseline infant RPs and follow-up RPs respectively BEPs in most models. **Conclusions**: Interventions should consider the partially mediating role of parenting stress, especially for the later development of BEPs. Research should aim to identify additional factors influencing infant regulatory problems and subsequent behavioral difficulties.

## 1. Introduction

Infant regulatory problems (RPs), defined as difficulties in self-regulation, are among the most common mental health problems in early childhood [1,2]. According to Papoušek’s model, symptoms of RPs are conceptualized as a triad comprising (i) problems in the child’s behavioral and emotional regulation, manifesting in symptoms such as excessive crying, sleeping and/or feeding problems, (ii) excessive demands on parents, and (iii) dysfunctional parent–child interaction resulting in failures of co-regulation [3]. Prevalence estimates differ significantly across studies due to variations in classification systems, guidelines, and populations. Among full-term, typically developing children, the prevalence of excessive crying ranges between 5% and 26% [4]. Sleeping problems affect approximately 10–33% of infants, and feeding problems occur in about 9% to 43% during the first three years of life [4,5,6,7,8,9]. Regarding co-occurrence, most children exhibit a single regulatory problem, whereas a substantial proportion show multiple problems. Specifically, according to a study by Cook et al. [7], 25.5% of children had one RP, 20.5% had two RPs, and 7.3% had three RPs.

RPs are often transient, but can persist from infancy, through toddlerhood to preschool years [10,11]. Persistent RPs in the first six months of life are associated, for example, with sleeping problems and multiple RPs at 18 months [7]. Moreover, robust evidence indicates that single, multiple, as well as persistent RPs are associated with later behavioral and mental health problems [11,12,13,14,15]. A recent meta-analysis found that children with RPs had a cumulative incidence of 23.3% for behavioral problems in childhood and showed significantly greater behavioral difficulties than healthy controls [14].

Parental factors play a central role in children’s emotional regulation and socio-moral development. Developmental models, including the Integrative Developmental Framework, propose that children’s regulatory capacities emerge within caregiver–child relationships, which form a primary context in which emotions are supported, shaped, and communicated [16]. Environmental sensitivity frameworks further suggest that caregiving disruptions have differential effects across children: those with higher sensitivity (e.g., sensory processing sensitivity) are more strongly affected by both adverse and supportive environments, increasing vulnerability under misattuned caregiving while also enhancing resilience in the context of responsive and warm care [16,17,18]. Consistent with this view, empirical research identifies parental sensitivity [19] and secure parent–child attachment [20] as important correlates of children’s emotional regulation and socio-moral development. Moreover, the association between emotional context and children’s emotion regulation strategies appears to be mediated by maternal sensitivity [19]. Supportive parental responses, such as guiding children’s emotional expression and regulation, are associated with positive developmental outcomes, whereas restrictive responses, including physical or verbal punishment, are linked to adverse outcomes such as an elevated risk of social and mental health problems [21,22,23]. Similarly, Papoušek’s triadic model of infant RPs highlights the role of parental factors, including excessive demands on parents [3]. Thus, parenting stress, which refers to the imbalance between parenting demands and available resources [24], is an important factor associated with infant RPs. First, RPs in infancy are commonly linked to increased parenting stress [25,26]. Georg et al. concluded in their cross-sectional study that more infant RP symptoms are associated with higher levels of maternal parenting stress [27]. Second, parenting stress is linked to later behavioral problems [28]. Research in early childhood indicates that higher parenting stress during infancy predicts greater mental health problems at age 3, with children of more highly stressed parents showing more than twice the odds of elevated difficulties on the preschool Strengths and Difficulties Questionnaire [29]. Third, it is assumed that parenting stress may influence the trajectory from early RPs to later behavioral difficulties. Evidence suggests that parental characteristics may partially mediate this relationship. For example, Smarius et al. [30] found that excessive infant crying doubled the risk of behavioral and mood problems at age 5–6, with maternal caregiving burden partially mediating this association. A German cohort study [31] found that the parenting stress subscales social isolation and bonding difficulties mediated the association between maternal depressive symptoms and infants’ excessive crying and sleeping problems.

However, research on the mediating role of parenting stress remains limited, and sleeping and feeding problems have largely been neglected in this context.

To sum up, early RPs are linked to elevated parenting stress, which is in turn associated with later behavioral difficulties. However, there is still limited research on whether and how parenting stress mediates the relationship between early RPs and subsequent behavioral and emotional problems (BEPs). Thus, we analyzed parent–child dyads at two time points aligned with their early pediatric check-up. We hypothesized

(1) That infant RP symptoms at baseline are related to infant RP symptoms, respectively toddler BEPs at follow-up.
(2) That parenting stress serves as a partial mediator in the association of RP symptoms between baseline and follow-up check-up.
(3) That parenting stress serves as a partial mediator in the association between RP symptoms at baseline check-up and behavioral problems at follow-up check-up.

## 2. Materials and Methods

### 2.1. Study Design

Patient collective of this retrospective analysis was derived from the larger German longitudinal study ‘Junge Familien & Corona’ (‘CoronabaBY’). Details are described elsewhere [32,33]. The study included a baseline assessment and a follow-up evaluation aligned with pediatric check-ups and was conducted from February 2021 to November 2022. Ethical approval of the ‘CoronabaBY’ study protocol was given by the committee of the Technical University (vote no. 322/20 S) and OSF registration was carried out.

### 2.2. Participants

Invitation of participants and data collection were carried out digitally via a smartphone app called ‘Meine pädiatrische Praxis’ (‘My pediatrician’) (www.monks-aerzte-im-netz.de), which is an established communication tool between pediatricians and patients. In a two-phase recruitment survey, invitations were sent to pediatricians (*n* = 300) in Bavaria. Of those, 24.3% (*n* = 73) gave informed consent via app and an invitation to participate in the ‘CoronabaBY’ study was delivered to their patients with children 0–3 years of age. All questionnaires were administered in German digitally via the app at baseline and follow-up. The baseline and follow-up assessments were linked to the next early pediatric check-up, known as “U-examination”, of the child. U-examinations are regular medical check-ups with pediatricians in Germany during infancy and childhood. Depending on the child’s age, either infant RPs or toddlers’ BEPs were assessed: The questionnaire for infant RPs ‘Crying, Feeding and Sleeping’ (CFS) [34] was linked to the invitation to U-examinations U4, U5, or U6 (=baseline), which are around the ages of 3, 6, and 12 months (defined as infants). Families with toddlers’ attending the check-up U7 or U7a, which is typically at the age of approximately 21 or 36 months (respectively defined as toddlers), received the ‘Strengths and Difficulties Questionnaire’ (SDQ) as an assessment tool for toddlers’ BEPs [35]. Because invitations to the respective U-examination were sent several weeks before the scheduled appointment, with timing varying across pediatric practices, infants may have been younger than 3 months at study entry. The present analysis was therefore restricted to families whose infants were assessed at baseline.

### 2.3. Measurements

#### 2.3.1. Sociodemographic Characteristics

Participants were queried on their relation to the child (mother/father/other), their age, country of origin, mother tongue, level of education, parental leave, parenting (single/with spouse), and financial situation. Furthermore, characteristics like child’s age, sex, siblings, and occurrence of chronical disease were asked. Parenting stress, infant’s RPs, and toddlers’ BEPs were assessed via the following standardized questionnaires.

#### 2.3.2. Parenting Stress

The analysis of parenting stress involved the German adaptation of the ‘Parenting stress index’ (PSI) from Abidin [24] ‘Eltern-Belastungs-Inventar’ (EBI) [36]. In this study, the parent domain was assessed. The score comprises the seven subscales ‘health’, ‘isolation’, ‘role restriction’, ‘parental competence’, ‘attachment’, ‘depression’, and ‘spouse relationship’. Twenty-eight questions were asked on a 5-point Likert scale (1 = strongly agree, 5 = strongly disagree). The total score range was from 28 to 140, which was converted to T-values. Cut-off values were set leading to three measures ‘not stressed’ (T-value < 60), ‘stressed’ (T-value = 60–69), and ‘strongly stressed’ (T-value ≥ 70) [36]. The parent domain was shown to have a good internal consistency (α = 0.93), and checking the retest reliability one year later displayed an r = 0.87 [36,37].

#### 2.3.3. Infant Regulatory Problems

Infant crying/whining/sleeping (CWS) and feeding problems were assessed at U4–U6 using the standardized questionnaire on crying sleeping and feeding (CFS) [34]. The questionnaire is a screening instrument designed to quantify symptoms of infant RPs during the first year of life. In this study, the subscale: ‘crying, whining, sleeping’ and ‘feeding’ were applied. In total, 38 questions were asked. Of those, three questions assessed excessive crying, 13 questions specifically analyzed ‘feeding’, and 22 questions focused on CWS. Excessive crying was analyzed with the Wessel criterion ‘rule of three’, i.e., crying for three hours a day on three days a week for three weeks [38]. Parents were asked to answer according to their last week with their infant and if for whatever reason this week was different than others, the last typical week. Answers were given on a 4-point scale (range from 1 to 4). Mean values of the scales were calculated. Higher scores indicate higher infant sleeping, crying, or feeding problems. Besides these scores, validated cut-off values were used for the scale ‘Crying, Whining, Sleeping’ (cut-off value: 1.84) as well as for ‘Feeding’ (cut-off value: 1.27). Dichotomous variables were created with subsequent cut-off values expressing ‘no problem’ or ‘noticeable problem’. The validity of the questionnaire was supported by high internal consistency for the total score (α = 0.90) and the subscales (α = 0.81–0.89), as well as by correlations with behavior diaries and clinical diagnoses [32,34].

#### 2.3.4. Toddlers’ Behavioral and Emotional Problems

Toddlers’ BEPs were assessed using the German version of Goodman’s Strengths and Difficulties Questionnaire (SDQ) [35]. In this study, the 20-item version for families with toddlers was administered at the U7 or U7a examination. Parents reported on their child’s behavior over the previous six months using a 3-point response scale (‘not true’, ‘somewhat true’, and ‘certainly true’). The SDQ total score comprises the subscales emotional symptoms, conduct problems, hyperactivity, and peer problems and ranges from 0 to 40. Based on the total score, cut-off values were applied to classify children as having ‘no problems’ (0–13), ‘borderline’ difficulties (14–16), or ‘noticeable problems’ (17–40). The instrument’s validity has been demonstrated through comparisons with similar questionnaires, such as the Child Behavior Checklist [39], and its internal consistency has been reported as α = 0.82 [39].

### 2.4. Statistical Analyses

Since questionnaires could only be digitally submitted if all items were completed, there were few missing values, mostly due to obvious misreporting of parental age. Some parents did not complete all EBI scales, as parents without a partner were unable to respond to the partnership-related items. Participants with missing data were excluded in the respective analysis. Differences in study variables between baseline, follow-up, and loss to follow-up were analyzed using *t*-tests for continuous variables and chi-square tests for proportions. Multiple linear regressions were performed with baseline CFS subscales as the predictor and follow-up CFS scores and the SDQ total score as outcome variables. The analysis of potential influencing factors was guided by theory and exploratory considerations based on previous research relevant to this study [33,40]. Accordingly, the following variables were entered as control variables: child’s age (in months) and sex (1 = male, 2 = female), child’s chronic disease (0 = no, 1 = yes), having siblings (0 = no, 1 = yes), parental education (0 = low, 1 = high), and financial situation (0 = low, 1 = high). For the linear regression models, independent variables were dichotomized as follows: Education status was dichotomized into high (university degree and high school diploma) and low (secondary and lower secondary school diploma). Financial status was also dichotomized into high (“large expenses possible” and “bigger additional expenses possible”) and low (“smaller additional expenses possible”, “little scope for additional expenses”, “additional expenses not possible”). Chronic disease or disability of the child was defined as any chronic disease (also allergy, hyperactivity) and/or disability. Potential mediation effects were analyzed using the SPSS PROCESS (Version 4.0) macro by Hayes with ordinary least squares regression to investigate whether the individual CFS scores at baseline predicted the CFS scores and SDQ total score at follow-up, and whether the direct path would be mediated by the EBI total score at baseline. To calculate confidence intervals and inferential statistics, bootstrapping with 5000 samples and employed heteroscedasticity-consistent standard errors were applied. The P_M_ (proportion of mediation) was calculated as the ration of the indirect effect to the total effect and can be interpreted as the proportion of the total effect that is mediated by the mediator, i.e., parenting stress (EBI total score) [41]. Requirements for calculating the multiple linear regression models and the mediation models were met. The estimation was based on a type 1 error of α = 0.01 for all calculations. Statistical analyses were carried out in IBM SPSS Statistics Version 29.0.

## 3. Results

### 3.1. Sample Characteristics

**Figure 1:**
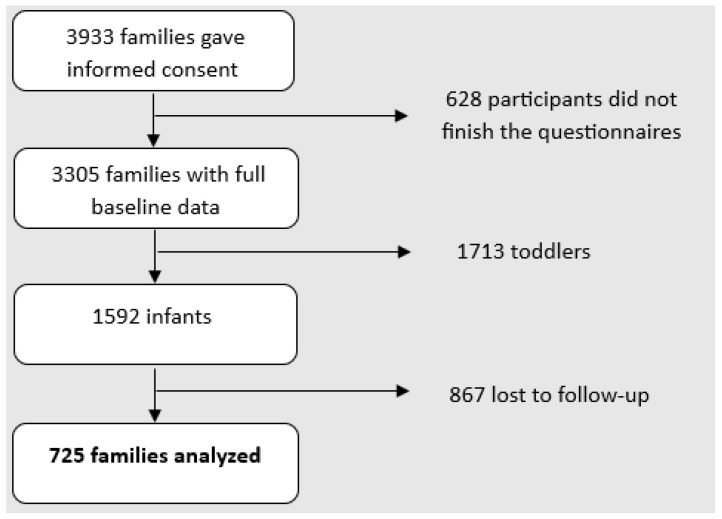
Flowchart.

Overall, 18,531 families with children aged 0–3 years received a push-message with the study invitation and further information. Of these, 3305 (17.8%) participants completed the questionnaires at baseline and 1292 at follow-up (loss-to-follow-up: 60.9%). The questionnaire CFS was administered to 1592 (48.2%) families at baseline. At follow-up, 725 families filled out the questionnaires, equivalent to a loss-to-follow-up rate of 54.5% (losses-to-follow-up *n* = 867) (Figure 1). Consequently, the initial study sample included the complete data of 725 participants (429 infants and 296 toddlers) at baseline and at follow-up. Sociodemographic characteristics are shown in Table 1. The vast majority of participants were mothers (94%), followed by fathers (5%). Average age for mothers was 33.0 years (SD = 4.2), and for fathers it was 34.4 years (SD = 4.5). Approximately 93% of the participants were born in Germany. About 63% of the sample had a high level of education, and a comfortable financial situation was reported by 58%. Mean infant age at baseline was 5.0 months (SD = 3.4). At follow-up, the mean age of infants was 9.5 months (SD = 2.52) and that of the toddlers was 19.6 months (SD = 1.59). The mean time interval between baseline and follow-up was 8.3 months (SD = 4.1, Min = 0.6, Max = 18.5), corresponding to an average of 35.6 weeks (SD = 17.7, Min = 2, Max = 80).

Participants retained in the study were more likely to be mothers, have German as their mother tongue, were more frequently currently on parental leave, and reported a more comfortable financial situation than those lost to follow-up (*p* < 0.05). Regarding infants, losses-to-follow-up were significantly older, had higher CFS ‘feeding’ scores, and similar CFS ‘CWS’ score. Moreover, parents lost to follow-up had a significantly higher EBI total score (*M* = 73.94) at baseline compared to the parents under study (*M* = 71.35, *p* = 0.012).

### 3.2. Children’s RP, BEP, and Parenting Stress

**Figure 2:**
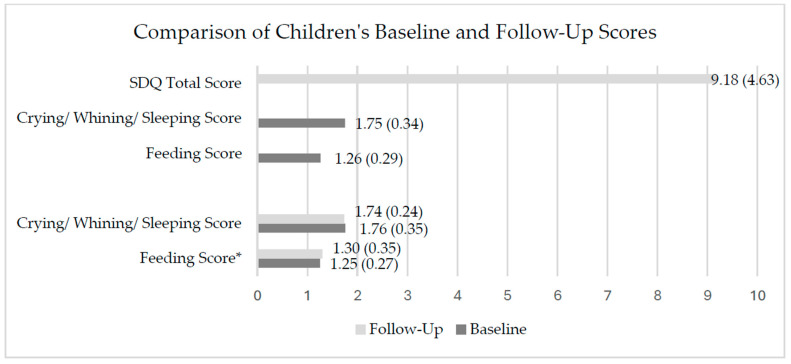
Comparison of children’s baseline and follow-up behavior scores (mean (SD)). * *p* < 0.05 for difference between baseline and follow-up.

Complete data on RPs at baseline and follow-up were available from 429 infants (Figure 2). At baseline, 33.6% (*n* = 144) of infants indicated noticeable feeding problems above the cutoff, 35% (*n* = 150) showed problems in ’CWS’, and 17.2% had both. Excessive crying following the Wessel’s criteria was noted in 4.4% of the infants. At follow-up, significantly more infants indicated feeding problems (*n* = 170, 39.6%, *p* = 0.002), ‘CWS’ problems were indicated in 31.5% (*n* = 135, *p* > 0.05), and 18.2% had both (*p* > 0.05). Excessive crying following the Wessel’s criteria was detected in 1.9% of the infants, which was significantly less than at baseline (*p* = 0.035).

At follow-up, complete data were received from 296 families with toddlers. At least ‘borderline’ emotional and behavioral problems were indicated in 17.3% (*n* = 51) of the toddlers (‘inconspicuous/normal’ = 82.8% (*n* = 245), ‘borderline’ = 10.5% (*n* = 31), ‘noticeable/abnormal’ = 6.8% (*n* = 20)).

At baseline, 37.1% of parents were stressed or strongly stressed (EBI total score = 71.5 (SD = 20.5), which increased to 41.2% (EBI total score = 75.0 (SD = 21.1, *p* < 0.001) at follow-up.

### 3.3. Linear Regression and Mediation Model

The multiple linear regression models yielded CWS at baseline to have the greatest effect on CWS at follow-up (Table 2). Likewise, feeding problems at baseline was the main predictor of feeding problems at follow-up. Regarding the prediction of the SDQ total score (i.e., toddler BEP), parenting stress at baseline had the highest effect size.

**Figure 3:**
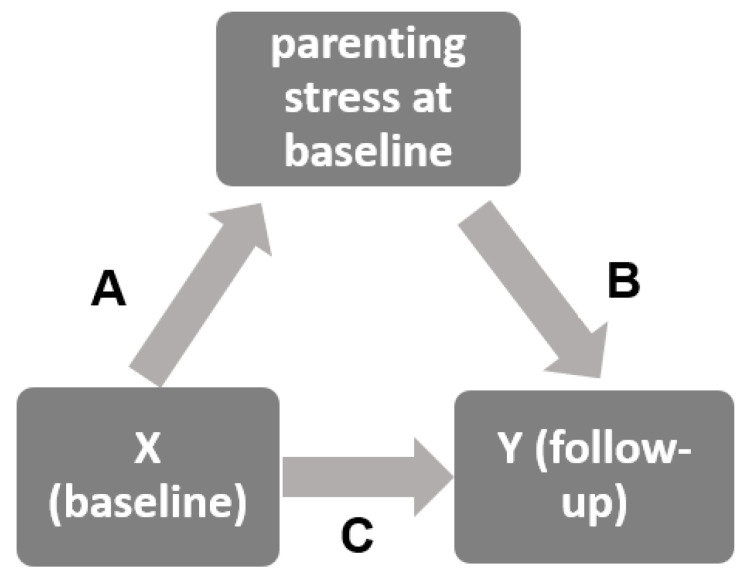
Basic mediation model. A, B, and C are depicted in Table 3.

The basic mediation model is presented in Figure 3. Results of the pathways are depicted in Table 3. Statistically significant total effects (C) from X on Y were evident in all analyses. After entering the mediator “parenting stress” into the models, all X variables (i.e., infants’ RP scores) were significantly associated with parenting stress. The mediator in turn predicted RPs and BEPs significantly in all but one case: Parenting stress did not predict sleeping and whining problems in Model 1. The indirect effect was significant in all but one model (No. 1). The proportion of mediation varied between 0.14 for the feeding model and 0.43 for the models with BEP as outcomes. This indicates that (1) the association between infants’ RP symptoms and (2) of the association between RP symptoms at baseline and BEP symptoms at follow-up were partially mediated by parenting stress.

## 4. Discussion

The present study on 725 parent–child dyads found an overall direct effect of infants’ RP symptoms at baseline on (1) infants’ RPs (i.e., CWS and feeding), and (2) toddlers’ BEPs at follow-up. These associations were partially mediated by parenting stress in most models.

The prevalences of 35% for CWS and almost 34% for feeding problems in our population are high, but still within the range of previously reported prevalences [4,5,6,7,8]. The present analyses found that elevated CWS and feeding problems at baseline significantly predicted those problems at follow-up. This is in line with several studies demonstrating a clear association between early and later symptoms of infant RPs [11,40,42].

In the present study, CWS problems did not change significantly from baseline to follow-up, whereas feeding problems increased significantly. This finding is consistent with a study by Olson et al. [42], who also reported higher rates of feeding problems in infants aged 8 to 11 months compared to younger infants aged 0 to 6 months. However, previous research identified heterogeneous trajectories of regulatory problems across the first years of life Schmid et al. found that sleep problems exhibit only short-term stability up to approximately 20 months of age, whereas feeding problems are more likely to persist). As our study included only two measurement points with varying follow-up time intervals, further longitudinal research with more follow-ups is warranted to replicate and extend these findings.

Furthermore, in the current study, infant RP symptoms significantly predicted later BEPs, which is in line with previous studies [13,43]. For example, Sidor and colleagues used the same scales for RP symptoms and parenting stress as in the present study and found that infants’ RPs at 6 months significantly predicted internalizing and externalizing problems at 36 months. Possible explanations for this association include impaired parent–child interaction and neurophysiological factors [43].

Parenting stress played a partial mediating role in the associations of baseline infant RPs with both later infant RPs and BEPs. The ratio of the indirect to the total effect (P_M_) suggests that 14% of the association between early and late feeding problems were explained by parenting stress, indicating a small mediation effect. Furthermore, parenting stress explained 24% of the effect of initial feeding problems on later CWS problems. Vice versa, parenting stress explained 33% of the effect of initial CWS problems on feeding problems, indicating a moderate mediation effect. Additionally, the effect of infant RP symptoms on later toddler BEP was also partially mediated by parenting stress, explaining a considerable part of the associations (42 to 43%). Similar findings were reported in the Dutch ABCD study, which identified maternal burden related to infant care and maternal aggressive behavior as mediators of the association between excessive crying and later behavioral and emotional problems (BEPs), assessed using the SDQ [30]. The results support the idea that parenting stress, accompanied by less sensitive, non-optimal parenting [44,45], may exacerbate infant crying, sleeping, and feeding problems through dysfunctional parent–child interaction patterns [3]. Although parenting stress was associated with CWS problems at the baseline, the mediation model using these problems as predictor and outcome was not significant, indicating that early parenting stress neither predicted nor mediated later CWS problems. Taken together with the partial mediation observed for other outcomes—and the small mediation effect for feeding problems—this suggests that factors beyond parenting stress may contribute more substantially to the persistence of child regulatory and behavioral difficulties. For example, Olsen et al. [42] reported that early RPs in infancy were a stronger predictor of later combined RPs than parental variables. Similarly, Sidor et al. [25] found that the effect of parenting stress disappeared when controlling for maternal depressive symptoms, and emphasized that child factors, such as temperament, may play a particularly important role. Importantly, as our analyses were based on correlations at the baseline, causal conclusions between parenting stress and child symptoms cannot be drawn. In the present study, these findings highlight the need to consider other variables such as child-intrinsic factors in addition to parenting stress when examining the persistence of infant regulatory and subsequent behavioral problems.

### Strengths and Limitations

The results must be interpreted in light of several strengths and limitations. To our knowledge, this is the first study to examine the potential mediation of parenting stress on the association between infant RP symptoms and later RP symptoms or BEPs in a large sample. Standardized questionnaires were used, which strengthens comparability. However, several limitations need to be taken into account. The CFS does not distinguish between isolated sleeping problems and crying/whining, and no distinction was made between single versus multiple RP symptoms, which may have concealed specific effects. Infant RPs were assessed via parent-reported continuous measures rather than clinical diagnoses, and higher parenting stress might increase their perception of problematic child behavior, which may overestimate the associations between parenting stress and child RP or BEP. The study design with only two measurement points precludes causal conclusions. However, assuming that RPs emerge independently from parenting stress allows us to conclude that parenting stress is a mediating factor on the association between RPs and BEPs. Additionally, children were not all the same age at follow-up due to linkage with routine pediatric check-ups, and intervals between assessments varied inter-individually. A short follow-up time interval might show rather short-term effects, whereas a longer time interval might show more manifested effects. Since the mean follow-up time ranged around 8 months, this relation should be balanced. Furthermore, the representativeness of the sample is limited. Participants were recruited via an app, which may have introduced selection bias. In addition, most participants were mothers, with German as their first language, a high level of education, and a comfortable financial situation. Including more fathers, family with migration background, or socially disadvantaged parents might have resulted in higher rates of parenting stress and maybe stronger effects [46]. Finally, compared to the population lost to follow-up, the sample had a better financial situation, less parenting stress, and more time with their children (i.e., no parental leave). Thus, the mediation might have been underestimated.

## 5. Conclusions

In our study, parenting stress emerged as a relevant factor that should be considered in the development of infant RPs and subsequent behavioral difficulties. Interventions targeting parenting stress may help to break the cycle of dysfunctional parent–child interactions that potentially maintain problem behaviors. Future research should aim to identify additional factors that influence the course of dysregulated behavior in children, in order to support the development of more targeted and effective interventions.

## Figures and Tables

**Table 1:** Sample characteristics at baseline.

TOTAL *N* = 725	%	*N*
Participants		
Mothers	94.1	682
Country of Origin: Germany	92.6	671
Mother Tongue: German	93.1	675
Currently on parental leave	88.1	639
Education		
University degree	41.0	297
High School Diploma	20.8	151
Secondary School Diploma	27.9	202
Lower Secondary School Diploma	8.1	59
Other	2.2	16
Financial Situation (before pandemic)		
Very large additional purchases possible	12.1	88
Large additional purchases possible	45.4	329
Small additional purchases possible	30.5	221
Very Small Additional Purchases Possible	4.6	33
No Additional Purchases Possible	0.3	2
Not Specified	7.2	52
Single parent	5.4	49
**Infants**		
Sex		
Male	49.4	358
Female	50.6	367
Chronic diseases	4.8	35
Siblings	43.0	312

**Table 2:** Linear regression models for analyzing the effects of infant and parental baseline variables on infant behavioral outcomes at follow-up.

Outcome Variables (Follow-Up)						
	Feeding Score (*n* = 408)		CWS Score ^1^ (*n* = 408)		SDQ Total Score ^2^ (*n* = 281)	
Coefficients (Baseline)	β	*p*-Value	β	*p*-Value	β	*p*-Value
CFS CWS ^1^	0.138	0.010	0.473	<0.001	0.201	0.002
CFS feeding	0.265	<0.001	0.110	0.017	0.142	0.017
EBI total score	0.130	0.015	0.050	0.302	0.238	<0.001
Child age	0.062	0.186	−0.044	0.303	0.051	0.366
Child sex ^3^	0.035	0.458	−0.119	0.005	−0.039	0.488
Chronic disease ^3^	−0.051	0.284	−0.043	0.328	0.080	0.155
Siblings ^3^	−0.096	0.047	−0.093	0.035	−0.003	0.959
Parental education ^3^	0.052	0.291	−0.035	0.436	−0.137	0.016
Financial situation ^3^	−0.068	0.164	0.009	0.838	−0.048	0.401

^1^ CWS score = CFS Crying, Whining, Sleeping score. ^2^ SDQ total score: indicates emotional and behavior problems. ^3^ Dichotomized.

**Table 3:** Results of the mediation models. Depicted are unstandardized coefficients and corresponding *p*-values for the pathways A, B, C (see Figure 2), indirect effect and proportion of mediation. Models were adjusted for child’s age, parental education, chronic disease, and siblings.

Model No.	X (T1)	Y (T2)	M	*n*	A	B	C (Total)	Indirect	P_M_^4^
1	CWS ^1^	CWS	Parenting stress at baseline	413	23.7969 (0.000)	0.0011 (0.1697)	0.5131 (0.000)	0.0264 (n.s.)	0.05
2	FP ^2^	CWS		413	24.3137 (0.000)	0.0035 (0.000)	0.3480 (0.000)	0.0839 *	0.24
3	CWS	FP		413	23.7969 (0.000)	0.0035 (0.0011)	0.2473 (0.000)	0.0823 *	0.33
4	FP	FP		413	24.3137 (0.000)	0.0027 (0.0068)	0.4754 (0.000)	0.0664 *	0.14
5	CWS	BEP ^3^		282	30.7634 (0.000)	0.0670 (0.000)	4.8331 (0.000)	2.0627 *	0.43
6	FP	BEP		282	26.0598 (0.000)	0.0741 (0.000)	4.6212 (0.000)	1.9310 *	0.42

* significant effect ^1^ CWS: infants’ crying/whining/sleeping problems, ^2^ FP: infants’ feeding problems, ^3^ BEP: toddlers’ behavior and emotional problems, ^4^ P_M_: Proportion of mediation.

## Data Availability

The data necessary to reproduce the analyses presented here are not publicly accessible.

## Abbreviations

The following abbreviations are used in this manuscript:

RPs	Regulatory Problems
BEP	Behavior and Emotional Problems
CFS	Questionnaire of Crying, Feeding and Sleeping
CWS	Crying, Whining, Sleeping
FP	Feeding Problems
EBI	Eltern-Belastungs-Inventar (Parenting Stress Inventory)
SDQ	Strengths and Difficulties Questionnaire
